# Maternal thyroid dysfunction and stress during pregnancy on ADHD risk in preschoolers: a retrospective study

**DOI:** 10.1007/s00787-025-02792-9

**Published:** 2025-06-23

**Authors:** Yongshen Feng, Junyan Li, Kris Yuet Wan Lok, Pui Hing Chau, Dali Lu, Jojo Yan Yan Kwok

**Affiliations:** 1 School of Nursing, Li Ka Shing Faculty of Medicine, The University of Hong Kong, Pokfulam, Hong Kong SAR, China; 2 Longhua District Maternity & Child Healthcare Hospital, Shenzhen City, Guangdong Province, China; 3 Psychology Department, Xiamen Fifth Hospital, Xiamen City, Fujian Province, China; 4 Centre on Behavioral Health, Faculty of Social Sciences, The University of Hong Kong, Pokfulam, Hong Kong SAR, China

**Keywords:** Attention-deficit/hyperactivity disorder, Maternal thyroid dysfunction, Maternal stress

## Abstract

Objective: To estimate the prevalence of attention-deficit/hyperactivity disorder (ADHD) in preschool children and to examine the relationship between ADHD and pregnancy-related factors across different trimesters in mothers. Method: The retrospective cohort study enrolled mother-child pairs from 20 kindergartens in the Longhua district, Shenzhen City, China. Adjusted Cox proportional-hazard models were used to estimate hazard ratio (HR) for the risk of pregnancy-related factors on ADHD. Results: Among 5602 mother-child pairs, 220 mothers (3.9%) were diagnosed with thyroid dysfunction, and 553 (9.9%) children aged 3–7 years were diagnosed with ADHD. In the adjusted model, children of mothers with thyroid dysfunction during pregnancy had a 54.1% higher risk of an ADHD diagnosis (95% CI: 1.050, 2.262) compared to those whose mothers did not have thyroid dysfunction. Higher maternal stress levels during the first trimester of pregnancy are associated with an increased risk of offspring developing ADHD. Compared to children whose mothers experienced significant stress in the first trimester of pregnancy, those whose mothers experienced moderate and minor stress had 36.3% and 42.9% lower risk of ADHD diagnosis, respectively (HR = 0.637, 95%CI: 0.487,0.834 for moderate; HR = 0.571 95% CI: 0.391, 0.833 for minor). However, stress during the second and third trimesters was not significantly associated with the risk of ADHD. Conclusion: The study demonstrates the need for early ADHD screening in preschoolers and continuous monitoring of maternal thyroid hormone and stress levels during pregnancy. It also emphasizes the importance of implementing preventive and management strategies as part of holistic care for both preschoolers and expectant mothers.

## Introduction

 Attention-deficit/hyperactivity disorder (ADHD) is a persistent neurodevelopmental disorder characterized by symptoms of inattention, hyperactivity, and impulsivity. Individuals with ADHD often experience comorbidities such as anxiety, depression, sleep problems, and oppositional defiant disorder [ 1 ]. Globally, the prevalence of ADHD in children aged 3 to 12 years is 7.6% [ 2 ]. In the United States, the prevalence ranges from 10.08 to 10.47% among children and adolescents from 2017 to 2022 [ 3 ] and the associated healthcare costs are substantial, estimated at $194 billion annually [ 4 ]. In contrast, the prevalence of ADHD is reported relevantly low in China (6.3%), from a systematic review of 67 studies involving 642,266 Chinese children and adolescents [ 5 ]. Additionally, a cross-sectional study of 6,719 Chinese students across 120 rural primary schools reported a prevalence rate of 7.5%, which aligns with the global average [ 6 ]. Although data on the annual increase in ADHD prevalence in China is lacking, the increased trends of ADHD prevalence rates and considerable financial and symptom burdens of ADHD observed in the United States (1997–2016) [ 7 ], underscore the importance of identifying potentially modifiable risk factors. Such identification is essential for enhancing our understanding of the etiology of ADHD and guiding interventions to enhance children’s health outcomes.

Thyroid hormones are crucial for normal brain development, and dysregulation of thyroid hormone levels during pregnancy can have detrimental effects on the neurodevelopment of offspring. Two systematic reviews found a significant association between an increased risk of ADHD in offspring and maternal hyperthyroidism (odds ratio, OR = 1.14) [8], as well as with congenital hypothyroidism [9]. This finding is further supported by a longitudinal study from Taiwan, China, which reported that prenatal hyperthyroidism significantly raised the risk of ADHD in offspring (OR = 2.23) among 330 mother-child pairs, compared to 1320 mothers without hyperthyroidism and their offspring [10]. Several studies have identified significant associations between objective indicators of thyroid function during pregnancy and the risk of ADHD. For example, elevated thyroid peroxidase antibody (TPOAb) levels are linked to a higher risk of hypothyroidism [11], and TPOAb positivity (TPOAb > 100 IU/ml) during pregnancy is significantly correlated with an increased risk of ADHD in offspring (OR = 1.6) [12]. Furthermore, a Finland birth cohort study of 9362 pregnancies and 9479 infants revealed a weak but significant association between increased maternal thyroid-stimulating hormone (TSH) levels in early pregnancy and ADHD symptoms in girls [13], necessitating further validation in different populations. However, most studies have been conducted in Western countries, with limited cohort studies reflecting the Chinese biological context. Additionally, existing literature relevant to this topic is roughly a decade old. Therefore, it is essential to investigate the relationship between maternal thyroid dysfunction and offspring ADHD in diverse populations and to update the data to reflect current trends.

Studies have examined the relationship between maternal mental health during pregnancy and the risk of ADHD in offspring. A systematic review of 58 studies found that maternal stress, anxiety, depression, prenatal stress, postpartum depression, and paternal depression were all associated with an increased risk of ADHD in offspring [14]. Additionally, a longitudinal cohort study in Norway indicated a significant link between maternal depression or anxiety and polygenic scores for ADHD in 14,539 pregnant mothers (OR = 1.15) [15], which was supported by another Finland cohort study [16]. At a more detailed level, maternal psychological status fluctuates across trimesters, with the third trimester typically exhibiting the highest level of anxiety [17]. Investigating how maternal stress during each trimester influences the risk of ADHD in offspring could guide targeted preventive strategies. However, few studies have specifically examined this association.

The Biopsychosocial Model offers a holistic framework for understanding the multifaceted nature of health and illness, emphasizing that the development of conditions (e.g., ADHD) cannot be attributed solely to biological factors, but also to social and psychological dimensions [18]. For children at risk for ADHD, maternal thyroid dysfunction and stress during pregnancy may be mutually influential. A review corroborates this, revealing that excessive stress is a key trigger for thyroid dysfunction, as elevated cortisol levels can inhibit pituitary gland function and reduce TSH secretion [19]. Exploring the combined impact of maternal stress, thyroid dysfunction, and sociodemographic factors on the risk of ADHD in offspring is therefore important. However, research examining the associations between these factors and childhood ADHD remains limited.

Guided by the Biopsychosocial Model, this study aims to bridge gaps by integrating maternal thyroid dysfunction during pregnancy as a biological factor and maternal stress during pregnancy as a psychological factor, while also accounting for the impact of social factors. Specifically, this study aimed to (1) investigate the prevalence of ADHD among preschoolers in urban Chinese kindergartens; and (2) examine how maternal thyroid dysfunction and stress during pregnancy affect the risk of ADHD in offspring. Drawing from the existing literature, we hypothesized that children born to mothers with diagnosed thyroid dysfunction and higher stress during pregnancy would have an increased risk of being diagnosed with ADHD.

## Methods

### Study design and participants

This retrospective cohort study used follow-up data from the Longhua Children Cohort Study database, which initiated in September 2021 with annual follow-ups, aims to investigate the impact of demographic characteristics, child’s birth and living environment, mother’s pregnancy, and family environments on children’s developmental functions, such as autism-like and hyperactivity behaviors [20, 21]. Mother-child pairs were recruited when they were enrolled in 20 kindergartens in the Longhua district, Shenzhen City, China. In 2022, follow-up data were collected through questionnaires administered to mother-child pairs, with the study protocol receiving approval from the Ethics Committee of a hospital in mainland China. All mothers endorsed the written informed consent following the Declaration of Helsinki.

We recruited participants based on the inclusion and exclusion criteria below. Inclusion Criteria: (a) Children aged between 3 and 7 years who are currently enrolled in preschools were included to avoid confounding with typical toddler behavior (e.g., short attention spans) under age 3 years and minimize recall bias in pregnancy exposure data for those over 7 years; (b) Mothers have basic reading and writing skills in Chinese and be able to communicate in Chinese; (c) Mothers are the biological mothers of the children and serve as the primary caregivers. Exclusion Criteria: (a) Children with confirmed diagnoses of severe psychiatric disorders, such as schizophrenia and paranoia; (b) Mothers who are undergoing treatment for life-threatening terminal disease (e.g., malignancies or severe cardiovascular diseases); (c) Mothers or children who have participated in other similar cohort studies within the past year to prevent biases related to repeated participation.

The rule of thumb for Cox regression models recommends a minimum of 10 outcome events per predictor variable [22]. In our study, the total number of predictors was 35, indicating that the number of ADHD cases (events) should exceed 350 to meet this criterion. Given the database contained over 5,600 participants and an ADHD prevalence of 6.3% from the literature [5], the number of events should well exceed the required 350 for the analyses, demonstrating that the sample size is sufficient.

### Measurements

#### Measurement of maternal thyroid dysfunction during pregnancy

A simple question measured maternal thyroid dysfunction during pregnancy: “Has the mother ever been diagnosed with thyroid dysfunction during pregnancy while pregnant with this child? " The answer options were “no” and “yes”. Mothers provided the answer when completing self-administered structured questionnaires.

#### Measurement of maternal stress during pregnancy

Maternal stress across pregnancy trimesters was evaluated using question sets of three questions: “Which of the following values best reflects your level of stress during the first trimester of pregnancy” “Which of the following values best reflects your level of stress during the second trimester of pregnancy?” and “Which of the following values best reflects your level of stress during the third trimester of pregnancy?“. Mothers were invited to complete this question set. Each question was scored on a five-point Likert scale, ranging from 1 (very minor) to 5 (very significant). Higher scores reflected higher levels of maternal stress during pregnancy. In our study, the Cronbach’s α coefficient for the question sets was 0.921.

#### Measurement of ADHD symptoms in preschool children

The core symptoms of ADHD were measured by the Chinese version of the Swanson, Nolan, and Pelham Rating Scale– Version IV (SNAP-IV) [23, 24]. Mothers were invited to complete the SNAP-IV, which is the most critical tool for ADHD screening and diagnosis [25]. The scale consists of two subscales with 18 items: inattention (items 1–9) and hyperactivity/impulsivity (items 10– 18), which align with the Diagnostic and Statistical Manual of Mental Disders 4th edition (DSM-IV) criteria for ADHD diagnosis [26]. Each item is rated by a 4-point Likert scale, ranging from 0 (not at all) to 3 (very much). Subscale scores were calculated by summing item scores, with higher scores indicating greater symptom severity. ADHD was categorized into three subtypes: inattentive, hyperactive-impulsive, and combined (both inattentive and hyperactive/impulsive). An ADHD diagnosis is indicated by an inattention score of 13 or higher and/or a hyperactivity/impulsivity score of 13 or higher. The Cronbach’s α coefficients of inattention and hyperactivity/impulsivity subscales for the Chinese population were 0.90 and 0.89, respectively [27]. In this study, the Cronbach’s α coefficient for inattention and hyperactivity/impulsivity subscales were 0.863 and 0.855 respectively.

### Covariates

Mothers of preschool children provided covariates information by completing self-administered structured questionnaires. Based on existing literature [28–30], the covariates in this study included children’s age (original values, months) and gender (male, female), prematurity (no, yes), height at birth (original values), weight at birth (original values), kindergarten numbers (1–20), class level in kindergartens (junior, middle, senior), maternal age at delivery (25 or younger, 26–35, 36 or older), maternal education (below junior college, junior college or above), gestational diabetes (no, yes), gestational hypertension (no, yes), and monthly household income (≤ 20, 000 yuan, > 20, 000 yuan).

### Statistical analysis

**Fig. 1:**
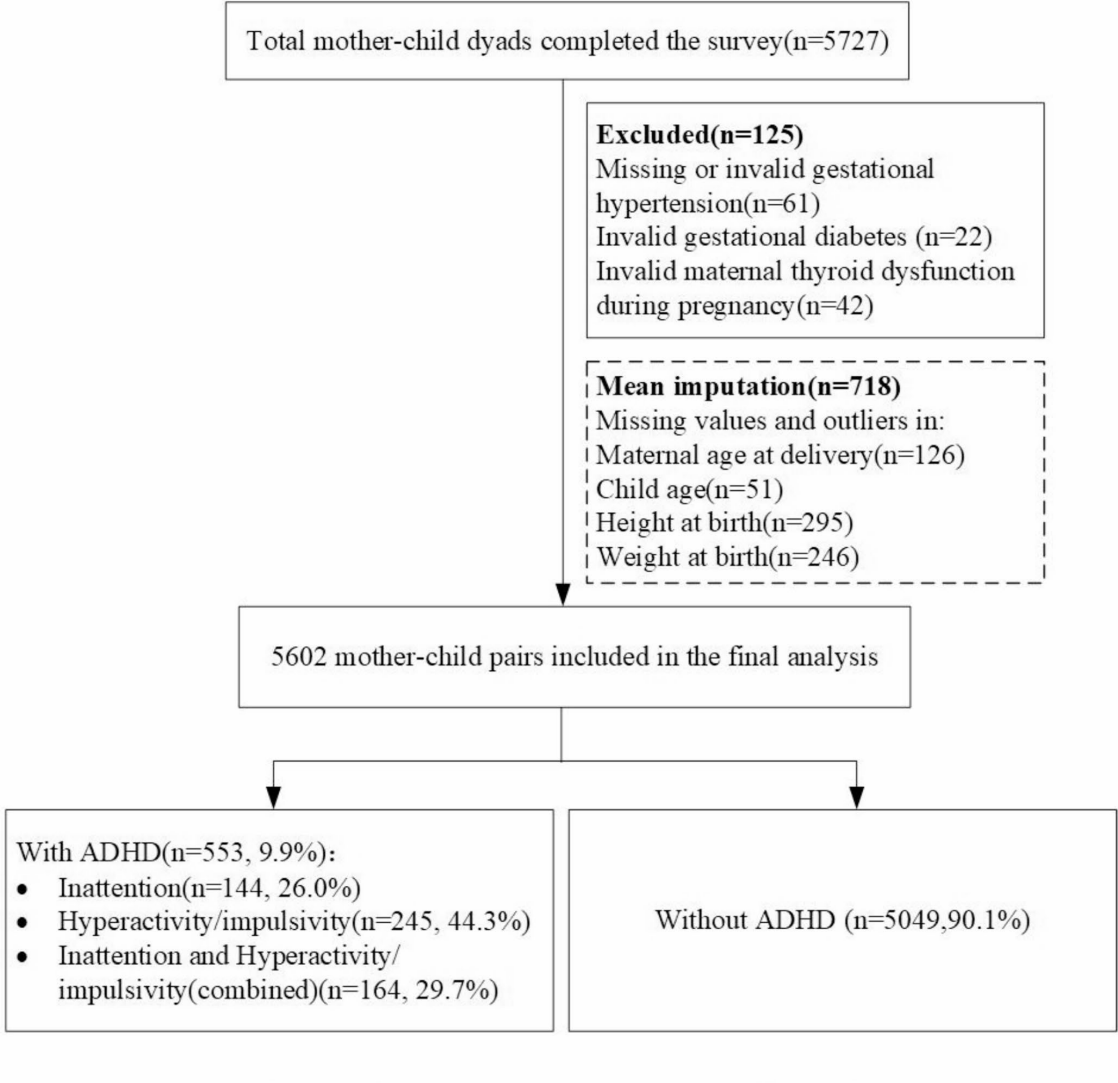
Flowchart of participant selection

Statistical analyses were performed by using the IBM SPSS software (version 27). Descriptive statistics of participants with and without ADHD are presented as the mean (standard deviation [SD]) for continuous variables and frequency (proportions) for categorical variables. Cases with missing categorical data (e.g., thyroid dysfunction and gestational hypertension) were excluded from the analysis (See Fig. 1). Missing continuous data were imputed with the mean score. Normality of continuous variables was assessed using visual inspection of histograms and Q–Q plots due to our large sample size (*n* = 5602). Two-sample independent t-tests and chi-squared tests were used to compare the child, maternal, and family characteristics of those with or without risk of ADHD.

We employed the Cox proportional hazards model to evaluate the associations between exposure to maternal thyroid dysfunction and stress during pregnancy with the risk of ADHD, with the time-to-event defined as the child’s age between 3 and 7 years [31]. The results are HR with 95% confidence intervals (CIs) and P-values. A P-value of < 0.05 (two-sided) was considered statistically significant. The main analysis assessed the risk of an ADHD diagnosis, with maternal thyroid dysfunction and stress during pregnancy as key exposure variables. Before fitting the model, we checked for multicollinearity by calculating the variance inflation factor (VIF) for all variables, including both the key exposure variables and covariates. The VIF values above the threshold of 5 indicated multicollinearity [32]. In the unadjusted model, maternal thyroid dysfunction and stress during pregnancy (across the first, second, and third trimesters) were tested. In the adjusted model, we added all the covariates mentioned above. We performed stratified analyses to compare ADHD prevalence between groups with and without thyroid dysfunction and among levels of maternal stress (Scores of 1–2 are minor, 3 is moderate, and 4–5 are significant.) during the first trimester.

To assess the robustness of the results, we conducted sensitivity analyses when sequentially removing each covariate one by one, and performed sensitivity analyses when only using the complete data set (i.e., data without missing values). The model was then refitted, and the HR, p-values, model fit statistics, and overall stability were compared with those from the original model [33].

## Results

In January 2022, we distributed 5,965 questionnaires and received 5,727, resulting in a response rate of 96.01%. After excluding 125 questionnaires due to reporting uncertainty, 5,602 mother-child pairs were included in the final analysis. Among 5602 children screened, 553 (9.9%) were diagnosed with ADHD, with the hyperactive/impulsive type being the most prevalent at 44.3%. Figure 1 shows the selection process of study participants.

Table 1 presents the demographic characteristics. All continuous variables approximately followed a normal distribution. Among 553 children diagnosed with ADHD, 348 (62.9%) were male. At diagnosis, the children’s mean age was 56.93 months (4.64 ± 0.87 years, range 3 to 7 years). Maternal thyroid dysfunction during pregnancy was diagnosed in 220 mothers (3.9%) of the total sample (*N* = 5602), including 29 (5.2%) in the ADHD group (*N* = 553) and 191 (3.8%) in the non-ADHD group (*N* = 5049). Mean maternal stress scores across the first, second, and third trimesters were 3.25 (0.95), 3.37 (0.85), and 3.30 (0.95), respectively. Further details are provided in Table 1.

**Table 1:** Characteristics of study population stratified by ADHD (*n* = 5602)

	ADHD (*N* = 553)	Non-ADHD (*N* = 5049)	Total(*N* = 5602)
**Maternal Characteristics**			
Maternal age at delivery (yrs.)			
25 or younger	141(25.5)	1007(19.9)	1148(20.5)
26–35	379(68.5)	3639(72.1)	4018(71.7)
36 or older	33(6.0)	403(8.0)	436(7.8)
Maternal education			
Below Junior College	247(44.7)	1947(38.6)	2194(39.2)
Junior College or above	306(55.3)	3102(61.4)	3408(60.8)
Gestational hypertension			
No	541(97.8)	4956(98.2)	5497(98.1)
Yes	12(2.2)	93(1.8)	105(1.9)
Gestational diabetes			
No	515(93.1)	4714(93.4)	5229(93.3)
Yes	38(6.9)	335(6.6)	373(6.7)
Thyroid dysfunction during pregnancy			
No	524(94.8)	4858(96.2)	5382(96.1)
Yes	29(5.2)	191(3.8)	220(3.9)
Monthly household income (CNY)			
≤ 20,000 Yuan	313(56.6)	2708(53.6)	3021(53.9)
> 20,000 Yuan	240(43.4)	2341(46.4)	2581(46.1)
Maternal stress in the first trimester	3.25 ± 0.95	3.54 ± 0.91	3.51 ± 0.92
Maternal stress in the second trimester	3.37 ± 0.85	3.63 ± 0.85	3.60 ± 0.86
Maternal stress in the third trimester	3.30 ± 0.95	3.58 ± 0.89	3.55 ± 0.90
**Children’s Characteristics**			
Age (Months)	56.93 ± 10.58	57.62 ± 10.37	57.55 ± 10.39
Gender			
Male	348(62.9)	2648(52.4)	2996(53.5)
Female	205(37.1)	2401(47.6)	2606(46.5)
Prematurity			
No	509(92.0)	4729(93.7)	5238(93.5)
Yes	44(8.0)	320(6.3)	364(6.5)
Weight at birth (kg)	3.25 ± 0.50	3.23 ± 0.49	3.23 ± 0.49
Height at birth (cm)	50.85 ± 2.87	50.82 ± 3.04	50.83 ± 3.02
Class level in kindergartens			
Junior class	162(29.3)	1370(27.1)	1532(27.3)
Middle class	187(33.8)	1800(35.7)	1987(35.5)
Senior class	204(36.9)	1879(37.2)	2083(37.2)

Abbreviation: ADHD, attention-deficit/hyperactivity disorder.

Note: Children’s age refers to the age at the time of the earliest diagnosis, which is the first point at which the ADHD was diagnosed.

**Fig. 2:**
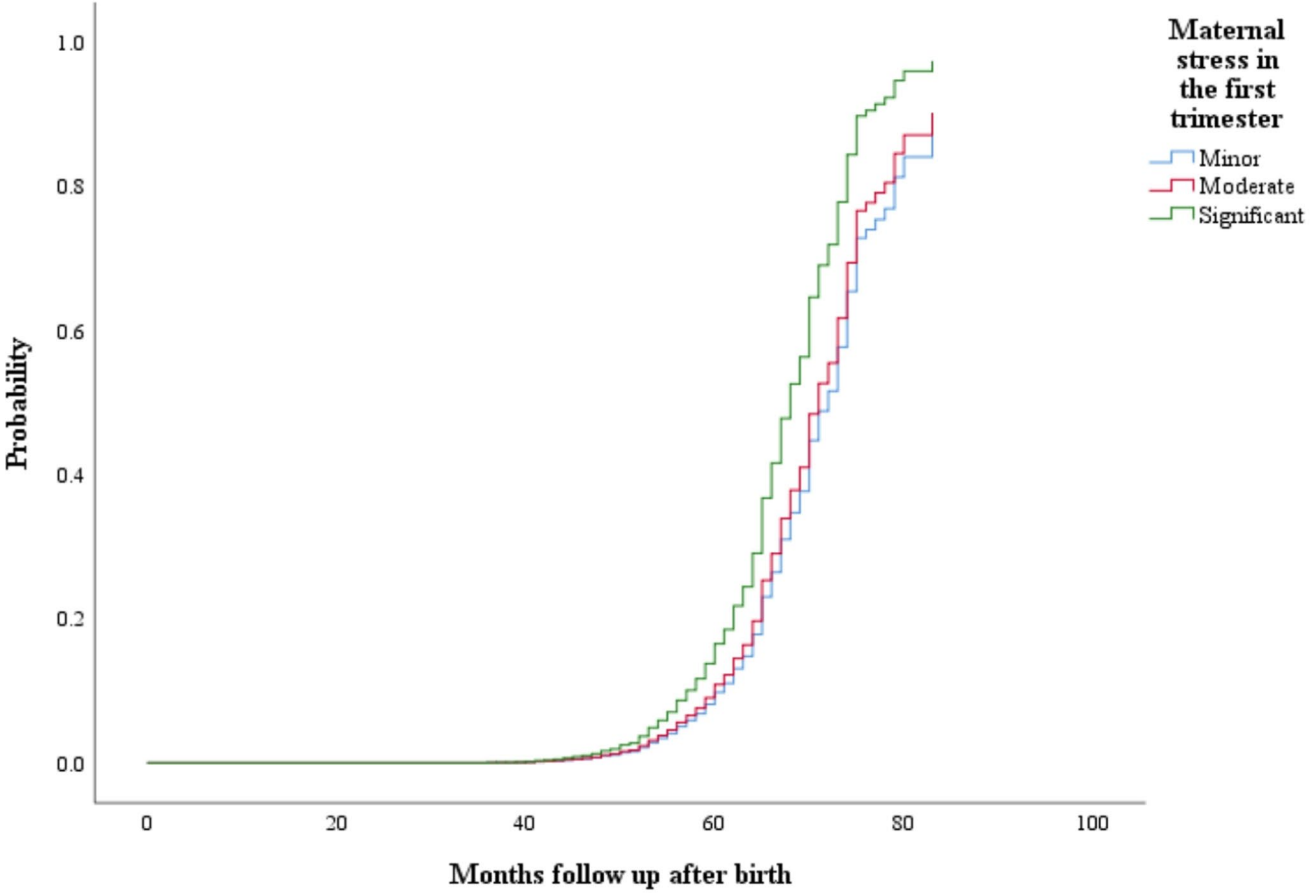
Cumulative prevalence of preschoolers’ ADHD diagnosis stratified by the maternal stress level in the first trimester of pregnancy

In Table 2, the Cox regression model results showed that higher maternal stress levels during the first trimester of pregnancy are associated with an increased risk of offspring developing ADHD (HR = 0.637, 95%CI: 0.487,0.834, in Table 2), while maternal stress in the second and third trimesters was not significantly associated with ADHD risk. To further understand the links between ADHD risk and maternal stress in the first trimester, we categorized maternal stress as a categorical variable (significant, moderate, and minor) in a rerun of the Cox regression analysis. In Table 3; Fig. 2, we found that compared to children whose mothers experienced significant stress in the first trimester of pregnancy, those whose mothers experienced moderate and minor stress had 36.3% and 42.9% lower risk of ADHD diagnosis, respectively (HR = 0.637, 95%CI: 0.487,0.834 for moderate; HR = 0.571 95% CI: 0.391, 0.833 for minor).

**Table 2:** Risk of ADHD by level of maternal stress in the first trimester (*n* = 5602)

	Unadjusted Model		Adjusted Main Model	
	HR (95% CI)	*P*	HR (95% CI)	*P*
Maternal thyroid dysfunction during pregnancy	1.758(1.206,2.562)	0.003	1.541(1.050,2.262)	0.027
Maternal stress in the first trimester (scores)	0.829(0.707,0.972)	0.021	0.851(0.726,0.998)	0.047
Maternal stress in the second trimester	0.988(0.801,1.219)	0.908	1.036(0.837,1.281)	0.747
Maternal stress in the third trimester	0.857(0.722,1.017)	0.078	0.865(0.728,1.027)	0.097

Abbreviations: ADHD, attention-deficit/hyperactivity disorder; HR, hazard ratio; CI, confidence interval

Maternal thyroid dysfunction during pregnancy: 0 = without, 1 = with

Maternal stress in the first, second, and third trimester: original values. Higher values indicated lower stress

Adjusted Main Model: Cox proportional hazards models were adjusted for maternal age at delivery, maternal education, gestational hypertension, gestational diabetes, monthly household income, child gender, prematurity, weight at birth, height at birth, kindergarten numbers (1–20), class level in kindergartens

**Table 3:** Risk of ADHD by level of maternal stress in the first trimester (*n* = 5602)

	Unadjusted Model		Adjusted Main Model	
	HR (95% CI)	*P*	HR (95% CI)	*P*
Maternal thyroid dysfunction during pregnancy	1.752 (1.203, 2.554)	0.004	1.541 (1.050, 2.262)	0.027
Maternal stress in the first trimester (scores)		0.003		0.004
4–5 (significant level)	1 (REF)		1 (REF)	
3 (moderate level)	0.640 (0.490, 0.836)	0.001	0.637 (0.487, 0.834)	0.001
1–2 (minor level)	0.546 (0.374, 0.799)	0.002	0.571 (0.391, 0.833)	0.004
Maternal stress in the second trimester	0.971 (0.798, 1.182)	0.771	1.025 (0.840, 1.250)	0.810
Maternal stress in the third trimester	0.855 (0.723, 1.011)	0.066	0.861 (0.728, 1.019)	0.082

Abbreviations: ADHD, attention-deficit/hyperactivity disorder; HR, hazard ratio; CI, confidence interval

Maternal thyroid dysfunction during pregnancy: 0 = without, 1 = with

Maternal stress in the first, second, and third trimester: original values. Higher values indicated lower stress

Adjusted Main Model: Cox proportional hazards models were adjusted for maternal age at delivery, maternal education, gestational hypertension, gestational diabetes, monthly household income, child gender, prematurity, weight at birth, height at birth, kindergarten numbers (1–20), class level in kindergartens

**Fig. 3:**
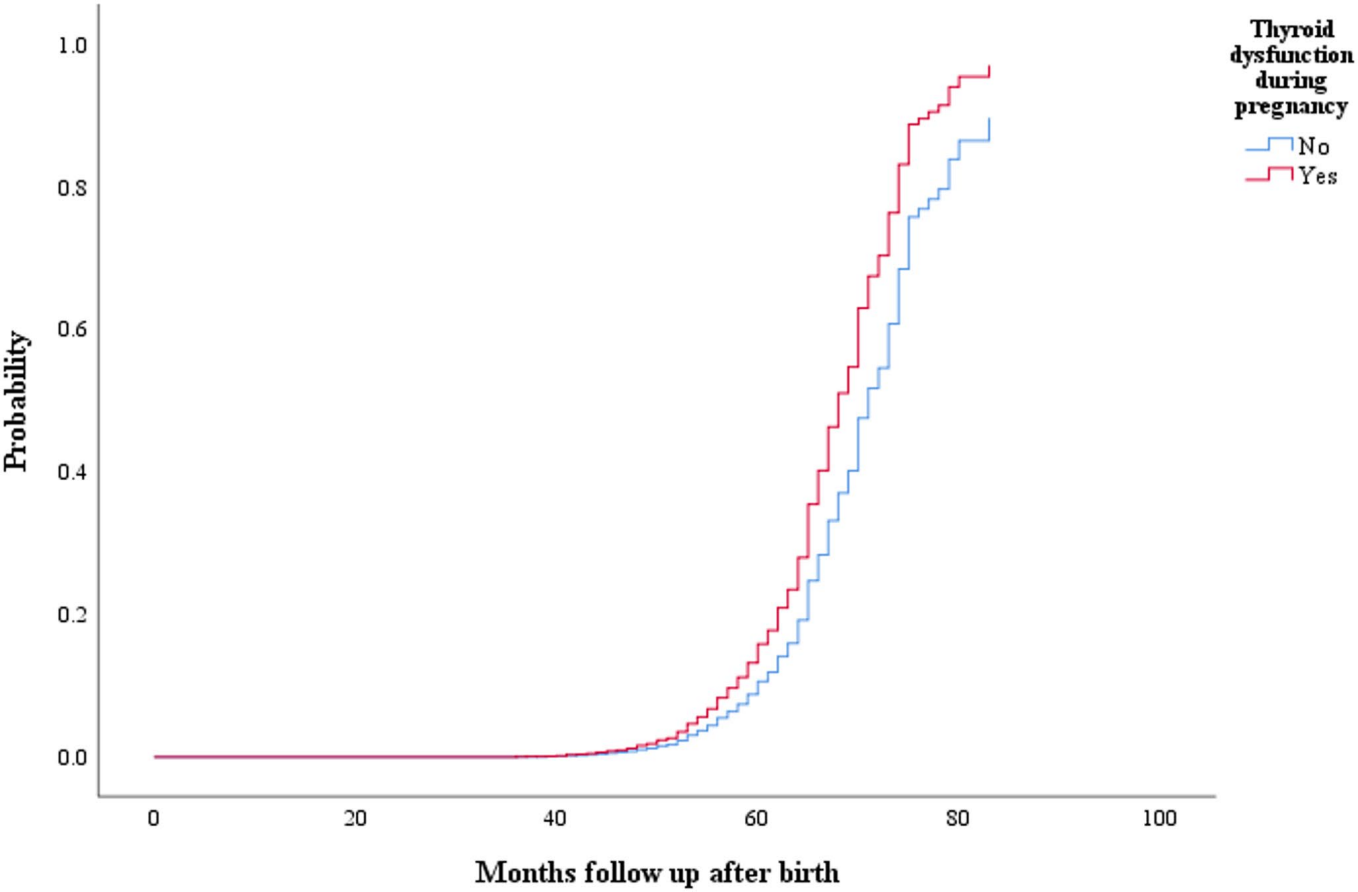
Cumulative prevalence of preschoolers’ ADHD diagnosis stratified by with or without maternal thyroid dysfunction during pregnancy

In Table 3, cox regression results indicated that the risk of ADHD diagnosis among children whose mothers had thyroid dysfunction during pregnancy was 75.2% higher (95% CI: 1.203, 2.554) compared to those whose mothers did not. After adjusting for covariates, the risk of ADHD remained 54.1% higher (95% CI: 1.050, 2.262) in children whose mothers had thyroid dysfunction during pregnancy (Table 2). Figure 3 illustrates the cumulative prevalence of preschoolers’ ADHD diagnosis associated with maternal thyroid dysfunction (present vs. absent). The data shows that the cumulative risk of ADHD increases with the child’s age between 3 and 7 years. Children whose mothers had thyroid dysfunction faced a higher risk of ADHD compared to those whose mothers did not, with the most pronounced difference observed between 60 and 84 months of age.

The collinearity diagnostics indicated that the VIF for all variables were below the threshold of 5, suggesting that multicollinearity was unlikely. The sensitivity analysis showed that HR for the primary exposure variables (maternal thyroid dysfunction and stress), along with P-values, model fit statistics, and overall stability, remained similar across models, indicating that the Cox proportional hazards model is robust to changes in covariate inclusion or data sets with or without missing data.

## Discussion

This is the first study to provide valuable insights into ADHD prevalence and associated pregnancy-related risk factors among preschoolers in the urban Chinese context, addressing a notable research gap. Our retrospective cohort study revealed that 3.9% of mothers were diagnosed with maternal thyroid dysfunction and 9.9% of children were at high risk for ADHD. The main findings also validated our hypothesis, demonstrating the effects of maternal thyroid dysfunction and stress during pregnancy significantly increase the risk of offspring ADHD, even after controlling for covariates. Furthermore, the analysis underscores the particularly significant impact of stress during the first trimester on ADHD risk, highlighting the critical need for early psychological screening and care in pregnancy.

This study identified that 9.9% of preschoolers were at high risk for ADHD, closely aligning with the prevalence rates in the US, which range from 10.08 to 10.47% [3], and exceeding the global prevalence of 7.6% [2]. Notably, the prevalence rate of ADHD among Chinese preschoolers from 20 kindergartens in our sample surpasses the 7.5% prevalence observed in school-age children from rural Chinese counties [6], and the 6.5% prevalence from a systematic review of 67 studies involving 642,266 Chinese children and adolescents [5]. One possible explanation for these discrepancies is the younger age of our sample (mean age of 4.74 /years) compared to the older age (mean age of 12.36 years) in Pang et al.‘s study. Additionally, our use of the SNAP-IV [24] as a screening and diagnostic tool may identify more potential cases than Pang et al.‘s study in which children have already received a formal ADHD diagnosis [6].

Maternal thyroid dysfunction during pregnancy was associated with an increased risk of ADHD (HR = 1.541), consistent with the findings from a meta-analysis and a longitudinal study in Taiwan [8, 10]. The meta-analysis identified 29 eligible studies and found a significant association between maternal hypothyroidism and an increased risk of ADHD (OR = 1.14) [8]. Similarly, a longitudinal study in Taiwan reported that prenatal hyperthyroidism significantly increased the risk of ADHD in offspring (OR = 2.23) after adjusting for demographic factors and maternal mental health disorders [10]. These findings support the role of maternal thyroid dysfunction as a potential risk factor for ADHD in offspring. This underscores the importance of maintaining thyroid function for both maternal and children’s health. Ge and colleagues further supported this by indicating that maintaining balanced TSH levels during gestation is essential for fetus neurodevelopment [8]. Two reasons may explain this significant association. Firstly, the thyroid hormones are critical regulators in the early development of the fetal brain, including the differentiation of neurons, oligodendrocytes, astrocytes, and microglia [34]. Furthermore, fluctuations in maternal thyroid hormone levels during pregnancy are associated with an increased risk of spontaneous miscarriage, stillbirth, and low birth weight infants, which may contribute to an increased risk of ADHD [35, 36]. However, treatment for thyroid dysfunction during pregnancy remains a controversial issue [37]. Our findings underscore the necessity of developing optimal preventive and management strategies for maternal thyroid dysfunction (e.g., hyperthyroidism and hypothyroidism) during pregnancy. For instance, levothyroxine treatment is encouraged to be utilized based on the TPOAb status and serum TSH levels [38].

Maternal stress across different pregnancy trimesters was a crucial factor in assessing the risk of ADHD in offspring. High maternal stress in the first trimester of pregnancy was significantly associated with a higher risk of ADHD, which partially aligned with previous studies [14–16], but contrasted with one study [39]. Compared to children whose mothers were under significant stress, children born to mothers with moderate and minor stress levels during the first trimester had a 36.3% and 42.9% decrease in ADHD risk, respectively. Some mothers may feel physically stressed as they adapt to early pregnancy reactions such as nausea and vomiting. Additionally, maternal stress can induce the secretion of cortisol and glucocorticoids, triggering inflammatory responses that may disrupt fetal hormone levels and contribute to atypical development [40, 41]. There are two potential explanations for this association. On the one hand, stress during pregnancy can also lead to concurrent depression and anxiety in mothers, which may lead to antidepressant impacts on ADHD risk [14, 42]. On the other hand, mothers with stress and depression may engage in unhealthy lifestyle behaviors, such as smoking and overeating, which can negatively impact prenatal neurobiological influences on ADHD risk [15].

We found no significant association between maternal stress during the second and third trimesters and ADHD risk, which aligns with findings from Andreasen et al. [43] but contrasts another study reporting a strong link [39]. These discrepancies may arise from differences in the assessment of stress (e.g., cortisol vs. Perceived Stress Scale) and ADHD (e.g., The Conners Comprehensive Behaviour Rating Scales vs. SNAP-IV). A possible explanation for the non-significant association involves the timing of fetal brain development and maternal protective mechanisms [44]. After 13 weeks of gestation, the maturing fetal hypothalamic-pituitary-adrenal axis and the placental 11β-hydroxysteroid dehydrogenase type 2 enzyme, which inactivates cortisol, may buffer the adverse effects of maternal stress [45]. Further research is needed to clarify these mechanisms and incorporate objective measures to better understand the link between prenatal mental health and fetal neurodevelopment.

### Strengths and limitations

This study has several notable strengths. Guided by the Biopsychosocial Model (George Engel,1977), the study deepens our understanding of ADHD’s etiology by concurrently examining the effects of physiological (thyroid dysfunction) and psychological (stress) factors during pregnancy, with social factors controlled as covariates. Moreover, a pioneering aspect is its identification of potential ADHD cases and associated pregnancy-related factors in a large sample of 5,602 preschoolers from 20 urban Chinese kindergartens. This large-scale, population-based design facilitates the acquisition of a more representative population sample. Additionally, our research contributed to the existing literature by demonstrating that maternal thyroid dysfunction during pregnancy was associated with a 54.1% increase in the risk of ADHD in offspring in urban China. Lastly, while previous studies have examined the link between maternal mental health and offspring ADHD risk, they have not specifically investigated the effects of stress across individual trimesters [14, 15]. Our study filled this gap by being the first to explore the association between maternal stress in different trimesters and the risk of ADHD in offspring. We found that maternal stress during the first trimester of pregnancy was particularly influential. Therefore, implementing preventive approaches to mitigate maternal psychological stress in the first trimester is vital for offspring neurodevelopment.

This study has several limitations. First, all outcome assessments relied on maternal self-reports, which might induce recall or social desirability bias. Future studies are suggested to consider clinician-reported data or laboratory tests (e.g., TSH levels, cortisol levels, and ADHD diagnostic data) obtained from medical records to enhance the objectivity of the findings. Second, our study data was collected from 20 kindergartens in a single region, so the generalizability of findings to other regions might be limited. Third, as the retrospective cohort design may suggest potential causal links between exposure and outcomes, it cannot provide definitive evidence of causality. Hence, well-designed longitudinal studies are warranted to strengthen causal inferences. Fourth, we employed non-validated questions to evaluate maternal stress throughout the pregnancy trimesters, and the omission of a ‘no stress’ category could have led to certain misclassifications. Although these questions provided valuable insights, the absence of a standardized and validated scale may compromise the comparability of our findings. Fifth, the absence of data on maternal antidepressant uses and postnatal psychological outcomes of mothers limited our in-depth understanding of pregnancy factors in the offspring’s ADHD development. Moreover, thyroid dysfunction was recorded only as a binary variable (yes or no); further research should examine its types, duration, and treatment. Future research is suggested to adjust for these potential confounders or biologically relevant covariates to more accurately examine associations.

### Implications

Here are some implications for clinicians and policymakers. Firstly, given the 9.9% prevalence rate of high-risk ADHD in offspring aged between 3 and 7 years, promoting early detection in kindergartens or during routine pediatric check-ups can enhance the outcomes for children with ADHD. Furthermore, it is essential to implement effective strategies for treating maternal thyroid dysfunction and maintaining thyroid hormone stability. Moreover, our research underscores the importance of preventing or mitigating maternal stress during pregnancy, particularly in the first trimester. Lastly, targeted ADHD screening for children born to mothers who experienced thyroid dysfunction or significant stress during pregnancy could enhance both detection rates and long-term outcomes in children and mothers. Consistent with the Biopsychosocial Model, our main findings underscore the importance of concurrently considering psychological and physical factors (such as thyroid dysfunction and stress during pregnancy) together. Therefore, it is advisable to offer family-centered screening and treatment programs, considering the family as a holistic unit of care, for preschoolers and mothers who have mental disorders or experienced thyroid dysfunction during pregnancy.

## Conclusion

This study identifies a 9.9% high-risk prevalence of ADHD among preschoolers, significantly associated with maternal thyroid dysfunction and stress in the first trimester of pregnancy. The findings emphasize the critical need for early screening of ADHD in kindergartens and the importance of monitoring maternal thyroid hormone levels and stress during pregnancy. To enhance long-term outcomes, it is recommended to provide holistic and preventive care to preschoolers and their mothers during pregnancy.

## Data Availability

The data that supports the findings of this study are available when contacting the corresponding author: Dr. Jojo Yan Yan KWOK.
