# Correction: Maternal thyroid dysfunction and stress during pregnancy on ADHD risk in preschoolers: a retrospective study

**DOI:** 10.1007/s00787-025-02834-2

**Published:** 2025-10-18

**Authors:** Yongshen Feng, Junyan Li, Kris Yuet Wan Lok, Pui Hing Chau, Dali Lu, Jojo Yan Yan Kwok

**Affiliations:** 1 School of Nursing, Li Ka Shing Faculty of Medicine, The University of Hong Kong, Pokfulam, Hong Kong SAR China; 2 Longhua District Maternity & Child Healthcare Hospital, Shenzhen City, Guangdong Province China; 3 Psychology Department, Xiamen Fifth Hospital, Xiamen City, Fujian Province China; 4 Centre on Behavioral Health, Faculty of Social Sciences, The University of Hong Kong, Pokfulam, Hong Kong SAR China

**Correction: European Child & Adolescent Psychiatry**

10.1007/s00787-025-02792-9

In the original version of this article, the equal contribution section was missing from this article and should have read “Yongshen FENG and Junyan LI are co-first authors, contributing equally to this work. Jojo Yan Yan KWOK and Dali LU are co-corresponding authors, contributing equally to this work”.

